# Plaie de l'artère sous-clavière gauche par un tournevis: à propos d'un cas

**DOI:** 10.11604/pamj.2014.18.75.4281

**Published:** 2014-05-23

**Authors:** Hicham Labsaili, Said Makani

**Affiliations:** 1 Service de Chirurgie Cardio-Vasculaire, CHU IBN ROCHD, Casablanca, Maroc

**Keywords:** Artère sous-clavière, chirurgie, détresse respiratoire, Subclavian artery, surgery, respiratory distress

## Abstract

Nous rapportons le cas d'un patient présentant une plaie de l'artère sous-clavière gauche suite à un traumatisme par un tournevis. Il s'agit d'une lésion rare mais grave, qui entraine souvent des complications neurologiques et respiratoires pouvant être mortelles. Dans les pays en voie de développement, la chirurgie classique à ciel ouvert reste de premier recours.

## Introduction

Les plaies de l'artère sous-clavière (ASC) sont peu fréquentes et constituent moins de 2% de l'ensemble des plaies vasculaires en milieu civil [1]. L'expérience clinique limitée, la difficulté d'exposition chirurgicale et la complexité anatomique de la région ont rendu la gestion de ce type de lésions très difficile. La mortalité opératoire varie de 5% à 30% et liée à l'hémorragie massive et rapide [2]. Nous rapportons le cas d'une plaie de l'artère sous-clavière gauche révélée par une paralysie du plexus brachial, une détresse

## Patient et observation

Un jeune patient de 20 ans a consulté aux urgences, après une semaine de son agression par un tournevis au niveau de la base gauche du cou. L'orifice d'entrée était petit et un point sur la peau a été fait. Après une semaine, le patient commence à avoir une voix rauque, un hématome augmentant de volume, un syndrome de Claude Bernard Horner, une paralysie totale de son membre supérieur gauche, cette dernière a motivé sa famille à consulter aux urgences.

A son admission, le patient présente un grand hématome de la région sus-claviculaire et carotidienne gauche, une dysphagie et un syndrome de Claude Bernard Horner. Une exploration angiographique a été demandée. Juste avant la réalisation de l'examen radiologique, le patient a fait une détresse respiratoire avec désaturation et cyanose. L'exploration radiologique a été annulée et le patient est conduit directement au bloc opératoire des urgences vasculaires.

### Technique opératoire

**Figure 1:**
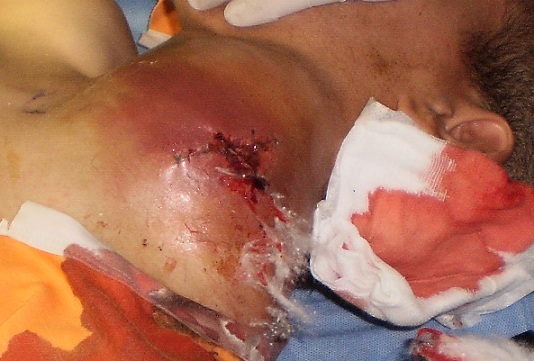
Montre l'hématome compressif rendant l'intubation très difficile

**Figure 2:**
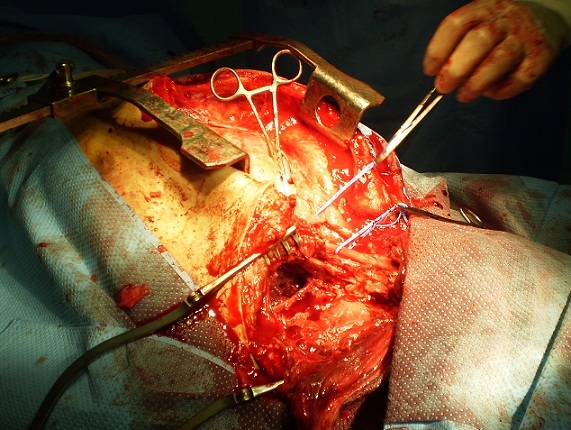
Sternotomie prolongée par une cervicotomie transverse pour contrôler la partie proximale de l'ASC

**Figure 3:**
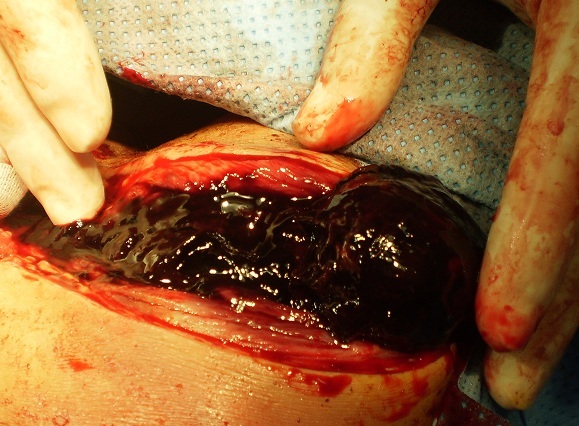
Évacuation d'un énorme hématome

Sous anesthésie générale et après une intubation trachéale très difficile, à cause de la déviation latérale de la trachée par l'hématome (Figure 1), une sternotomie médiane a été faite, ouverture du péricarde, dissection et exposition de l'artère carotide gauche et de l'artère sous-clavière gauche, mise des deux vaisseaux sur lacs et contrôle de leurs parties proximales (Figure 2). Extension de l'incision en sus-claviculaire, clampage de l'origine de la sous-clavière gauche, évacuation de l'hématome (Figure 3), et puis une exploration chirurgicale soigneuse a été faite pour trouver la plaie vasculaire. Un test de déclampage rapide a localisé la lésion.

**Figure 4:**
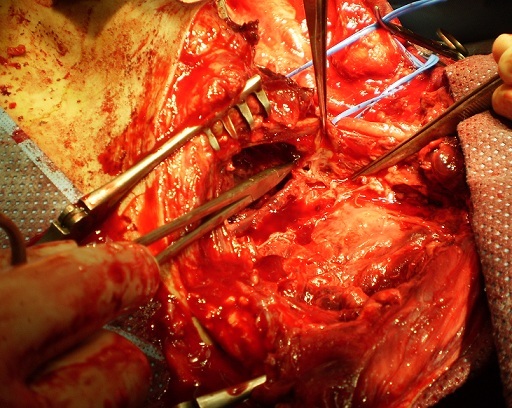
Plaie localisée après test de déclampage

C'est une plaie de 5mm au niveau de la partie proximale de l'ASC gauche (Figure 4), qui a été réparée par un surjet de monobrin 6/0.l'hémostase a été contrôlée ainsi que le pouls huméral homolatéral. Le patient a été fermé après mise en place de deux drains médiastinaux, un drain pleural gauche et deux drains dans la région cervicale gauche. Les suites post opératoires immédiates ont été marquées par une transfusion de 4 culots globulaires, 6 PFC et une éxtubation à H6.

Durant son hospitalisation, les signes cliniques du syndrome de Claude Bernard Horner ont régressés, il n'a plus de voix rauque, mais le patient garde toujours la paralysie de son membre supérieur, ce qui a nécessité une rééducation dans une autre structure. Le suivi post-opératoire après 3 mois a montré une récupération progressive de la motricité du membre qui n'est pas encore totale.

## Discussion

Les plaies pénétrantes de l'ASC continuent à poser un challenge chirurgical, elles sont rares et de présentation clinique très variable.il s'agit le plus souvent de plaies par armes blanches. Elles sont potentiellement graves avec un risque hémorragique, un risque respiratoire par hématome compressif et un risque neurologique par possibilité de compression du plexus brachial [3]. Les signes d'atteinte vasculaire sont un saignement actif rapidement mortel, des signes d'hypoxie et la présence d'un hématome. Dans notre cas, vu la petite taille de la plaie, le saignement était à minima mais continu, ce qui a causé un grand hématome compressif, responsable des signes cliniques chez le patient. Par contre, il n'y avait pas des signes d'ischémie du membre supérieur homolatéral vu la circulation collatérale [4].

Les explorations angiographiques sont très importantes afin de déterminer la tactique opératoire, la voie d'abord et la possibilité de faire un traitement endovasculaire chez certains patients [5]. Dans notre observation, nous n'avons pas pu réaliser aucune exploration vu l'aggravation respiratoire rapide.

Plusieurs techniques opératoires ont été décrites pour la gestion des plaies pénétrantes de l'ASC [6]. Une combinaison d'une sternotomie médiane à une cervicotomie transverse donne une excellente exposition de l'origine de l'ASC. L'abord supra-claviculaire peut être utile chez des patients avec des plaies du côté gauche. Si un long segment de l'ASC est endommagé, on peut faire une cléidectomie pour une meilleure exposition.

La réparation vasculaire peut se faire directement par des points séparés ou un surjet si on a une petite brèche, sinon, l'interposition d'un greffon veineux ou d'une prothèse est utilisée, avec une préférence du greffon veineux afin de prévenir une infection sur prothèse, vu le contexte du traumatisme qui se fait dans la majorité des cas par des armes blanches souillées.

Les innovations de la radiologie interventionnelle et les nouvelles techniques endovasculaires peuvent considérablement changer la gestion des traumatismes de l'ASC, mais malheureusement, nous ne disposons pas d'un plateau technique dédié à cette activité, ce qui nous conduit dans la plupart des cas de faire une chirurgie conventionnelle à ciel ouvert.

## Conclusion

Les plaies de l'ASC sont rares, mais graves. Ces lésions engendrent une mortalité et une morbidité importantes. Les manifestations cliniques sont l'hémorragie massive et l'hématome compressif. Dans notre cas, c’était l'hématome compressif qui était responsable de la symptomatologie. Dans les pays en voie de développement, ne disposant pas de moyens d'endovasculaire, la chirurgie à ciel ouvert reste la seule solution.
